# Towards a mission‐oriented approach to cancer in Europe: an unmet need in cancer research policy

**DOI:** 10.1002/1878-0261.12452

**Published:** 2019-02-01

**Authors:** Julio E. Celis, Manuel Heitor

**Affiliations:** ^1^ European Academy of Cancer Sciences Danish Cancer Society Research Centre Copenhagen Denmark; ^2^ Minister for Science, Technology and Higher Education Portugal

**Keywords:** cancer, cancer continuum, mission‐oriented approach, network of centres, scientific, social and economic impact

## Abstract

Today, cancer is a significant challenge for society, healthcare systems and the growing number of affected patients and their families. This article argues that new paradigms and conditions for responsible science and innovation policy across the European Union (EU) require (i) the collective action of Research & Development institutions, (ii) a system approach to health systems, higher education and patient organizations, and (iii) new initiatives to encourage international cooperation across an enlarged Europe; no single country can successfully fight the disease(s) on its own. Recently, a cancer mission was proposed (Celis and Pavalski, 2017), the origins of which are rooted in the continuous efforts of the research community, cancer patient organizations, member states and the European Commission during nearly two decades to address the fragmentation and lack of coordination of European cancer research; these efforts led to the creation of Cancer Core Europe and Cancer Prevention Europe, consortia aimed at linking therapeutic and prevention geometries. Ultimately, the platform/infrastructure will be composed of networks of Comprehensive Cancer Centres and cancer research centres across Europe to reach the critical mass of expertise, patients and collaborative portfolio of projects that are necessary to promote science‐driven and social innovations in the era of personalized (precision) cancer medicine. Employing a mission‐oriented approach to achieve the goal of ensuring a long life expectancy for three out of four cancer patients by 2030 is likely to have a particularly positive impact on the way European citizens’ value science and knowledge. It will change the lives of many families across Europe and beyond and should be oriented to ensure that Europe is at the forefront when it comes to quality of life. It is our collective responsibility to ensure that not a single person or region in Europe is left behind.

**Abbreviations**
CCC: Comprehensive Cancer Centre
CPE: Cancer Prevention Europe
EACS: European Academy of Cancer Sciences
ECCO: European CanCer Organisation
EC: European Commission
ECRA: European Cancer Research Area
EP: European Parliament
ERA: European Research Area
FP: Framework programme
UNESCO: United Nations Educational, Scientific and Cultural Organization

## 1 Introduction

This review provides a brief description of the steps leading to a potential cancer mission in Horizon Europe (Celis and Pavalski, 2017). The mission states that ‘by combining innovative prevention and treatment strategies in a sustainable state‐of‐the‐art virtual European cancer centre/infrastructure, it will be possible by 2030 to achieve long‐term survival of three out of four cancer patients in countries with well‐developed healthcare systems. In the long‐term, primary prevention will change the increasing cancer incidence. Furthermore, the proposed concerted actions will pave the way to handling the economic and social inequalities in countries with less developed systems. It will also ensure that in the long‐run, science‐driven and social innovations reach patients across the healthcare systems in Europe’. The mission is rooted in continuous efforts by the research community, cancer patient organizations, member states and the European Commission (EC) during nearly two decades to address the fragmentation and lack of coordination of European cancer research. These activities led to the creation of pan European platforms/infrastructures for translational cancer research, which include Cancer Core Europe (Eggermont *et al*., 2014) and Cancer Prevention Europe (CPE; Forman *et al*., 2018), consortia aimed at linking therapeutic and prevention geometries. Ultimately, the platform/infrastructure will be composed of networks of Comprehensive Cancer Centres (CCCs) and cancer research centres across Europe to reach the critical mass of expertise, patients and collaborative portfolio of projects that are necessary to promote science‐driven and social innovations in the era of personalized (precision) cancer medicine.

Making the potential mission a reality will require collective action across all of Europe to guarantee three basic conditions, namely (a) full open access to knowledge and data; (b) enlarged participation and effective engagement of academic clinical centres throughout Europe; and (c) the necessary relevance in terms of citizen engagement through the commitment of all national and regional governments across Europe. Furthermore, it must address the gap between science, society and policy, as well as ensuring the active involvement of the cancer research, cancer health care and cancer patient communities at all stages of policymaking, in order to align specific scientific and diversified local issues into an overall strategy with practical relevance to all European citizens at large.

Above all, the unique relevance of a mobilizing process towards an inclusive cancer mission in Europe is associated with its likely positive impact on the way European citizens believe in science and knowledge. We should note that some 50 years after John Ziman launched the discussion on Public Knowledge (Ziman, 1968) and 40 years after his work on grasping the significance of scientific knowledge in Reliable Knowledge (Ziman, 1978), one must understand the nature of science as a complex whole. In Real Science (Ziman, 2000), we are reminded that ‘science is social’, referring to ‘the whole network of social and epistemic practices where scientific beliefs actually emerge and are sustained’. Thus, an adequately defined cancer mission can provide a unique collaborative framework to contribute towards this goal and should be regarded as a critical step in promoting the scientific culture of European citizens at large. It is in this context that the prospects for a successful cancer mission for Europe should be considered, with a realistic perspective of our common path and recent past.

## 2 Thinking with history: the European Research Area (ERA) and its impact on translational cancer research

### 2.1 ERA and the creation of the European Cancer Research Area (ECRA)

Establishment of the European Research Area (ERA), a vision championed by Commissioner Philippe Busquin in the year 2000, placed science at the core of the European knowledge society (https://www.pbl.nl/sites/default/files/cms/publicaties/Towards_a_European_Research_Area.pdf). Progress in basic science was deemed as necessary as innovation, priming scientists to accept their societal responsibility, joining forces, building and organizing communities, and providing evidence‐based advice to inform policy. An impressive outcome of these activities was the establishment of the European Research Council in 2007 (Celis and Gago, 2014; Gannon, 2017; König, 2017), partly due to the efforts of the basic research community that spoke with a single voice to support research of the highest quality and rally researchers from most basic disciplines to participate in the ERC debate. Today, the ERC has grown to be the flagship of the EU Framework programmes (FPs; https://erc.europa.eu/about-erc/mission), and basic research has become the engine that fuels innovation.

Even though the ERA covered all areas of science, it was apparent that Europe did not deliver the treatments and diagnostics expected by healthcare professionals and citizens; as such, in 2002, Commissioner Busquin proposed, with the support of the European Parliament (EP), the creation of European Cancer Research Area (ECRA) at the ‘Towards greater coherence in cancer research’ conference (http://europa.eu/rapid/press-release_SPEECH-02-408_en.htm). At the time, there was a pressing need to bring basic knowledge into clinical practice, and creating a common joint strategy to meet the increasing burden posed by the disease was considered crucial. Thanks to the support of the EP, cancer became one of the priorities in FP6, and for the first time, an FP could explicitly support clinical research (Van de Loo *et al*., 2012). Concluding the Conference, Commissioner Busquin told the cancer community ‘ECRA will be what you make of it’, a simple, but powerful reminder that galvanized researchers and clinicians and incited them to join forces and become engaged in cancer research policy issues.

### 2.2 Addressing the fragmentation of European cancer research: establishment of Cancer Core Europe and Cancer Prevention Europe (CPE)

#### 2.2.1 The Eurocan + Plus project: implementing closer cooperation across borders

In 2004, Commissioner Busquin, with the support of the EP, set up a working group to identify ways of better coordinating cancer research in Europe; specifically, the group was asked to identify areas and research topics where there was a certain level of activity in the member states that could benefit from improved coordination at the EU level. Based on the recommendations of the working group as well as further discussions with the cancer community (Saul, 2008; Ringborg and Tursz, personal communication), the EC funded the Eurocan + Plus project in 2005 (lead by Peter Boyle) within the frame of the specific programme ‘Integration and Strengthening of the ERA’ in the domain Life Sciences, Genomics and Biotechnology for Health. Implementing closer cooperation across borders, together with the development of an overall strategy for cancer research at the European level, was expected to be cost‐efficient and facilitate the development of significant scientific advances and their delivery to citizens across Europe. Barriers to collaboration needed to be identified and ways sought to encourage the development of collaborations in the member states in order to speed up cross‐border partnerships. The project, which involved all major stakeholders, proposed in 2008 the creation of a platform for translational cancer research composed of interlinked cancer centres with shared infrastructures and collaborative projects to facilitate rapid advances in knowledge, and their translation into improved cancer care (http://ecancer.org/journal/2/full/84-eurocan-plus-report-feasibilitystudy-for-coordination-ofnational-cancer-research%20activities.php). It also emphasized the importance of CCCs, patient‐focused entities that connect research and education with care and prevention, and that link research with the healthcare system. At that time, a European accreditation methodology for CCCs was launched (Saghatchian *et al*., 2008; see also other articles in this issue). CCCs integrate every step in the cancer research–care continuum from basic/preclinical to early clinical, late clinical and outcomes research.

At the end of the Eurocan + Plus project in 2008, the FP6 had ended, and FP7 had already started, thus leaving a gap that needed consideration if the translational research platform proposal was to become a reality. FPs do not allow for continuity, and as a result, other options needed to be explored to transform the diffuse idea into a desirable goal for policymakers.

#### 2.2.2 The ‘Stockholm Declaration’: a paradigm shift in cancer research

On 6 November 2007, just before the end of the Eurocan + Plus project, the directors of 16 leading European cancer centres met in Stockholm to define the Platform concept, and to show their commitment they signed the ‘Stockholm Declaration’, openly stating their intention to join forces, share resources and expertise, and coordinate their funding (Ringborg, 2008). The ‘Stockholm Declaration’ signalled a paradigm shift in cancer research, which was catalysed by the dedication and collective vision of basic and clinical researchers agreeing to use a coordinated and concerted approach to work towards the implementation of a platform for translational cancer research by bringing together CCCs. The platform would aim to (a) harmonize and share infrastructures and competencies; (b) define and coordinate specific areas for research collaboration; (c) improve training and mobility of researchers; (d) attract young researchers from all over the world and retain talent in Europe; (e) provide the pharmaceutical and biotechnological industries with strategic partnership, and (f) accelerate translational research and drive innovation in Europe for the benefit of patients (Ringborg, 2008).

Several informal meetings took place in 2008 to coordinate and prioritize the activities of the Stockholm group. The first steps towards making the ‘Stockholm Declaration’ a reality took place at a meeting held at the United Nations Educational, Scientific and Cultural Organization (UNESCO) headquarters in Paris in 2008: ‘Turning the Stockholm Declaration into Reality: Creating a World‐class Infrastructure for Cancer Research in Europe’, jointly sponsored by the Danish Cancer Society, the Initiative for Science in Europe and UNESCO. The Conference was attended by 150 scientists, clinicians, policymakers, managers, patient organizations and representatives from industry. At this meeting, former Commissioner Busquin reminded the audience that ‘scientists must frame the platform challenge in a way that it targets the specific responsibilities of the EC as separate from the national responsibilities’ (Brown, 2009). Furthermore, he urged the cancer community to act quickly ‘as there were financial perspectives to be discussed in 2009’.

A very encouraging outcome of the Conference was the fact that the cancer community was perceived as a well‐structured group and a reliable partner/stakeholder for planning future research activities, including priority setting for future calls within the framework programmes. At this point, connecting with FP7 was on the horizon!

#### 2.2.3 Bridging the gap between cancer research and policy

At the UNESCO meeting, it became evident that to bridge the gap between science and policy it was necessary to engage large scientific organizations to sustain policy activities. Given the multidisciplinary composition of its members, broad international representation, large congress and focus on the patient, the European CanCer Organisation (ECCO) was deemed as being centrally positioned to integrate the cancer community and to provide a forum for debating European cancer policy issues that may benefit patients as well as society as a whole. Towards this end, ECCO created the Science Policy Committee in 2008, supported by well‐known policymakers as advisors (Philippe Busquin, Jose Mariano Gago, Frank Gannon, Federico Mayor Zaragoza and Peter Lange) (Celis and Gago, 2014). Furthermore, in 2009 ECCO created the European Academy of Cancer Sciences (EACS) to advise and support the organization in its quest to become the driving force of oncology in Europe and a significant player in structuring and developing ERA. ECCO also created the Oncopolicy Forum to nurture and inspire discussions on policy issues (Fricker, 2010).

#### 2.2.4 The EuroCanPlatform Network of Excellence: making the platform for translational cancer research a reality (Cancer Core Europe and CPE)

As a result of the Eurocan + Plus project recommendations, as well as the support from cancer organizations, the cancer community and policy advisers, the EurocanPlatform Network of Excellence, led by Ulrik Ringborg, was funded by FP7 in 2011. The project engaged 23 European cancer centres in 11 member states and five large scientific organizations (eCancer.eu, ECCO, ECPC, EORTC and OECI), with the task of creating a translational cancer research platform to promote innovation in prevention, early detection and therapeutics, with a focus on personalized medicine.

One of the main achievements of the EurocanPlatform was the creation of Cancer Core Europe in 2014 (Eggermont *et al*., 2014; see also the article by Eggermont *et al*., 2019), an accomplishment lead by A. Eggermont and O. Wiestler that applied a bottom‐up approach to initiate the process. Furthermore, in the spirit of the ‘Stockholm Declaration’, each of the participating centres allocated their own resources. The guidance and support of the late Jose Mariano Gago – former Portuguese Minister of Science, Technology and Higher Education during the various phases of the EurocanPlatform Consortium, including the creation of Cancer Core Europe, was instrumental in this endeavour.

Cancer Core Europe is a patient‐centred legal structure consisting of seven large cancer centres – most of them CCCs – working in partnership to address the whole cancer research–cancer care continuum (Eggermont *et al*., 2014). The critical mass of the infrastructure is substantial, with around 60 000 newly diagnosed cancer patients, 300 000 treated patients and 1 200 000 patient consultations annually. Moreover, more than 1500 clinical trials are being conducted at the centres. Cancer Core Europe is open to the addition of new centres; in the first instance, core centres will link with other CCCs and centres, with the aim of expanding contacts to all EU member states. In time, there must be at least one CCC in every EU member state to ensure that they link to national centres and reach out and interact closely with clinical centres concerning quality of care, innovation and research collaboration (see also articles by Eggermont *et al*., 2019, and by Berns, 2019).

Following the initial success of Cancer Core Europe, C. Wild from the International Agency for Research on Cancer organized a meeting in Lyon on the 6–7 July 2015 to explore the possibility of creating a similar consortium in prevention (CPE). Today, CPE is a reality, and it is composed of leading research organizations across Europe (Forman *et al*., 2018). Cancer Core Europe and CPE link the therapeutic and prevention geometries and will provide a virtual infrastructure that, in concert with other networks of cancer centres, will serve as a hub and an engine to coordinate and optimize joint cancer research efforts across Europe. It will also facilitate rapid advances in knowledge and their translation into better cancer treatment, care and prevention.

## 3 Bridging the cancer community efforts through H2020 and beyond

In 2015, Commissioner Carlos Moedas established three strategic priorities to deal with the challenges to the Research and Innovation policy: Open Innovation, Open Science and Open to the World (3'Os; https://era.gv.at/object/news/1893). Given that the 3'Os are interlinked, Julio E. Celis and Dainius Pavalski, members of the Open Science group of the Research and Innovation Science Experts HLG that advised the Commissioner and the Commission (https://ec.europa.eu/research/openvision/index.cfm?pg=expert-groups-rise), proposed that in addition to addressing the 3O's independently, it was critical to single out case studies of specific disciplines/research environments where these priorities could develop in accord to tackle a major societal challenge. Cancer Core Europe, being a legal entity, and with its significant critical mass of expertise, resources and patients, and its potential to innovate and engage in science diplomacy provided a unique example of how the 3O's could develop and progress in accord (https://publications.europa.eu/en/publication-detail/-/publication/15e2ff8d-c525-11e8-9424-01aa75ed71a1).

In July 2017, the ‘Lamy Group’ appointed by the Commission to maximize the impact of EU Research and Innovation Programmes suggested a mission‐oriented approach in FP9 (now Horizon Europe) to address global challenges (http://ec.europa.eu/research/evaluations/pdf/archive/other_reports_studies_and_documents/hlg_2017_report.pdf). In response to this proposal, and encouraged by the Commission, Julio E. Celis and Dainius Pavalski, with the support of leading cancer researchers, put forward a cancer mission based on the activities and aims of Cancer Core Europe and CPE (Celis and Pavalski, 2017).

The cancer mission was thoroughly discussed at the first Gago Conference on ‘European Science Policy’ – centred on cancer research in Europe and policy perspectives – that took place at the i3S in Porto, Portugal, on 14 February 2018 under the auspices of the Portuguese Agency for the Promotion of Scientific and Technological Culture – Ciência Viva, and with the support of the Portuguese Ministry for Science, Technology, and Higher Education (https://www.gagoconf.org/1st-edition/). Closing the meeting, Commissioner Carlos Moedas confirmed his strong support for a mission‐oriented approach to cancer in Horizon Europe (https://ec.europa.eu/commission/commissioners/2014-2019/moedas/announcements/first-gago-conference-european-science-policy_en).

On 7 June 2018, the Commission presented the Horizon Europe proposal, which included Research and Innovation missions within Pillar 2 (Global Challenges and Industrial Competitiveness). Missions were expected to have a scientific, social and economic impact; examples of missions could ‘range from the fight against cancer, to clean transport or plastic free oceans’ (http://europa.eu/rapid/press-release_IP-18-4041_en.htm). Encouraged by this development, the EACS, now an independent legal entity with a focus on science policy (https://www.europeancanceracademy.eu/), urged the ‘EU and its member states to formally launch a mission in cancer to boost and streamline the cancer research continuum in Europe’ (Adami *et al*., 2018). The cancer mission should develop in parallel with science policy and foresight, and the EACS has requested the newly established science policy committee to strengthen its activities to ensure that science‐driven and social innovations reach patients across healthcare systems in Europe (https://www.europeancanceracademy.eu/http-eacs-peterbourne-co-uk-science-policy-committee).

The mission, as well as its societal impact, was also discussed at the 2nd Gago Conference ‘Science, society and policy towards a Europe of knowledge: On the role of science engagement in Horizon Europe’ that took place in Vienna on 21 September 2018.

On 28 September 2018, the Competitiveness Council decided to include missions and large partnerships in the legal text of Horizon Europe and asked for closer involvement of member states in their strategic planning. On 15 October, at an informal lunch that took place at the Berlaymont Building in Brussels, the Council discussed preliminary ideas concerning missions based on a nonpaper presented by the Commission. Five potential mission areas, including curing paediatric cancer by 2030, were addressed. This development came as a surprise to the cancer community considering that focusing on 0.6% of all cancer patients will severely compromise the scientific, social and economic impact of such a mission (Ferlay *et al*., 2018).

Shortly after, the broad cancer mission (Celis and Pavalski, 2017) was discussed at a Conference that took place at the Vatican ‘A mission‐oriented approach to cancer in Europe: Boosting the social impact of innovative cancer research’ on 15 November 2018. The Conference, jointly organized by the EACS and the Pontifical Academy of Sciences (https://www.europeancanceracademy.eu/user_uploads/files/EACS%20PAS%20Vatican%20conference%20Nov%202018.pdf), was attended by researchers, clinicians, policymakers, diplomats, representatives of cancer leagues and patient organizations. A significant outcome of the meeting was the general agreement among the participants that the cancer community is well prepared to tackle a mission on cancer that should be broad and not restricted to paediatric oncology as put forward earlier by the Commission (see the European Academy of Cancer Sciences, 2019).

On 30 November, the Commission officially proposed five missions to the Competitiveness Council, including a mission on paediatric cancer. On 17 December 2018, however, it was agreed that the paediatric cancer mission should be replaced by a general cancer mission (https://sciencebusiness.net/framework-programmes/news/eu-governments-grapple-research-missions-and-partnerships).

## 4 Building new horizons for Europe: a collective effort for the years to come

The conclusion of the current 8th EU FP for Research and Innovation (Horizon 2020) in 2 years time, together with the emerging debate associated with the impact of Brexit, calls for a research and innovation policy formulation in Europe that adequately strengthens open and knowledge‐based cohesion platforms across Europe, promotes opportunities for precompetitive research and adds value through translational research. High priority should also be given to understanding the relevance of research with regard to quality of life.

We argue that a cancer mission in Europe will help reduce the current gap between science and policy, and this is necessary to achieve the above goals and the target of ensuring a long life expectancy for three out of four cancer patients by 2030 across Europe. The latter will require the active involvement of the cancer research, cancer health care and cancer patient communities in policymaking, in order to align specific scientific and diversified local issues into an overall strategy with practical relevance to all European citizens at large. As mentioned earlier, the EACS is committed to support the mission and is ready to provide evidence‐based advice to underpin policies that allow solutions and inform society of the benefits of research (Adami *et al*., 2018). In addition, patient organizations are willing to support the mission by engaging in sustained policy activities (see article by De Lorenzo and Apostolidis, 2019).

### 4.1 Establishing a clear vision across Europe

Promoting such a cancer mission across Europe is a critical condition for effectively implementing an inclusive process oriented towards the continuum of knowledge production and diffusion, encompassing all stages from basic to translational and market‐oriented research. The future EU FP for Research and Innovation should also act against the unidirectional migratory flows of skilled people from the peripheries to the centre of Europe, promoting brain circulation as well as advanced education and employment of skilled human resources all over Europe. A cancer mission can effectively spur these goals!

In particular, the promotion of knowledge‐based European added value and health‐related quality of life based on cancer needs requires persistent actions to foster precompetitive and translational collaborative research and innovation, together with multinational and fully European collaborative research laboratories, involving public and private stakeholders all over Europe, as well as further internationalizing knowledge and innovation networks. This should be assessed by research excellence, research openness and effective institutional collaboration at a European level.

Our basic postulate is that an adequately defined mission approach to research and innovation will facilitate a sizeable societal engagement, and this requires fostering research to improve public health and health systems across entire Europe. Ageing of the population, together with increasing requirements to better establish routines for the treatment of cancer (cancer is an example of the main chronic diseases) and new opportunities for implementing forms of personalized medicine, requires the promotion of clinical and translational research in the health sciences across Europe.

However, building a collective action across Europe to foster new horizons for European research and innovation with a strong European identity through a mission approach also requires that the following underlying conditions be met:

First, fully open access to knowledge and data for cancer treatment, care and prevention to guarantee additional and specific funding and assessment schemes of collaborative ventures between hospitals, health‐related research institutions, and schools of medicine, nursing and health technologies. This must always be implemented and assessed while taking into consideration research excellence, in addition to ensuring an active effort across all of Europe to share excellence in cancer treatment, care and prevention.

Second, increased participation and active engagement of academic clinical centres throughout Europe, in a way that allows the cancer mission to effectively help implement networks of CCCs representing different research geometries, together with a gradual and continuous enlargement of Cancer Core Europe and CPE. To achieve this goal, there must be at least one CCC in every EU member state to ensure that they interact closely with surrounding clinical centres with respect to quality of care, innovation, education and research collaboration. The ultimate goal should consider interlinked cancer research centres across all of Europe, with shared infrastructures and collaborative projects to facilitate rapid advances in knowledge and their translation into better cancer treatment, care and prevention.

Last, but not least, the required relevance regarding engaging citizens at large through the commitment of all national and regional governments across Europe to achieve the mission target. It must align specific scientific and diversified local issues into an overall strategy with practical relevance to all European citizens at large. Science museums and centres throughout Europe should be engaged and help to promote scientific culture in association with cancer treatment, care and prevention in schools and throughout society.

In other words, the uniqueness of a cancer mission for Europe relies on its ability to promote a quite diversified set of institutions, very often strongly affected by paths of cooperative interests, as well as to spur a new era of science–society relationships.

Continuous articulation at the European level for an integrative and mobilizing cancer mission will also require specific actions aimed at aligning diversified European funds (research and innovation as well as structural funds) and national funding sources. This will be necessary to (a) support infrastructures for cancer research (clinical and translational) in addition to clinical trials; (b) provide adequate incentives to undergo clinical research at the EU and national levels; and (c) support translational and clinical research professional careers with the necessary career development patterns at regional and national levels across Europe. These items should be considered in the context of engaging all regional and national governments.

## 5 Looking forward to a transforming process for Europe: relevant next steps

Our research together with the emerging debate in Europe about the cancer mission (see the European Academy of Cancer Sciences, 2019) suggests that broadening the social basis for knowledge‐based activities in cancer treatment and prevention, as well as strengthening the top of the research system leading to knowledge production at the highest level, should be considered together with the increasing need to foster intermediaries with society and the economy at large (Heitor and Horta, 2014). It requires a focus on the advanced qualifications of skilled people and teaching staff throughout the entire education system and its links with society. The latter is a continuous, long‐term process that requires a clear understanding of the role played by science–society relationships, beyond the currently dominating policies of thinking about science through short‐term, demand‐driven economic development issues.

An important lesson learned through analysis of the evolution of research and innovation across Europe in the last two decades is the need to create the conditions required for strengthening institutions and to form the necessary critical masses to engage in high‐quality research activities (Heitor *et al*., 2015). Following on from some of the issues raised by Ziman (1978, 2000) and, later by Ernst (2003) and Ernst and Kim (2002), one critically important institutional issue refers to the training of doctoral students and young scientists (see also article by Ernberg, 2019). These individuals should be provided with core competencies that help them to become successful researchers while preparing them with ‘transferable skills’ for the job market outside of academia, including the health system. The issue is increasingly relevant for Europe and the opportunities launched through a cancer mission should be considered in the three following lines of discussion:

First, it requires adequate public funding to train and attract skilled people and a teaching body, making use of proper research environments, including those with translational and clinical capacities. It must also consider the necessary funds across Europe to spur forms of international academic and scientific cooperation oriented towards the research training of young scientists and future physicians.

Second, at an institutional level, attracting skilled people and staff to strengthen translational and clinical research can be fostered by establishing a full EU network of CCCs, with at least one CCC in every EU member state (see also other articles in this issue). Our research shows that international academic and scientific cooperation seems to emerge as a major shaping factor for development at an unprecedented level to address these issues (Heitor, 2015a). Leading CCCs in Europe are now operating internationally, addressing not only local patients and research needs, but increasingly developing new types of institutional arrangements that can contribute to enhancing translational and clinical research capacity transnationally, benefiting economic and social progress in Europe at large (Heitor, 2015b).

Third, knowledge is a cumulative process, depending in the long‐run on the widespread disclosure of new findings. David (2007), among others, has originally shown that open science is properly regarded as uniquely well suited to the goal of maximizing the rate of growth of the stock of reliable knowledge (as defined by Ziman, 1978). As a result, CCCs in Europe could behave as ‘open science’ institutions and provide an alternative to the intellectual property approach to tackling difficult problems in the allocation of resources for the production and distribution of information on cancer treatments, care and prevention.

Also, the analysis suggests that major tasks should be conducted at a European‐wide level in association with the need to promote periodic research assessments of CCCs in Europe (see also articles by Berns, 2019; Oberst, 2019). This will have a significant impact on the building‐up of capacities and institutions throughout Europe (Heitor, 2015b) and should be implemented in association with a properly defined cancer mission.

Our research clearly shows that the co‐evolution of human capital formation and research capacity in its various forms (i.e. academic, translational and clinical) is critical to promote the absorptive capacity that regions and countries throughout Europe need to acquire to learn how to use science to effectively improve quality of life. In this context, a cancer mission is a key policy instrument to build new horizons for Europe. The target of achieving long‐term survival of three out of four cancer patients by 2030 is only 12 years ahead of us. This requires that collective effort be taken throughout and across entire Europe to build new trusted relationships between European citizens at large and science.

## Author contributions

Both authors contributed to the writing of this article.

## Conflict of interest

The authors declare no conflict of interest.

## References

[mol212452-bib-0001] Adami O , Berns A , Celis JE , de Vries E , Eggermont A , Harris A , zur Hausen H , Pelicci G and Ringborg U (2018) European Academy of Cancer Science‐position paper. Mol Oncol 11, 1829–1837. 10.1002/1878-0261.12379 PMC6210050 30241109

[mol212452-bib-0002] Berns A (2019) Quality‐assured research environments for translational cancer research. Mol Oncol 13, 543–548. 10.1002/1878-0261.12441 PMC6396364 30628170

[mol212452-bib-0003] Brown H (2009) Turning the Stockholm declaration into reality: creating a world‐class infrastructure for cancer research in Europe. Mol Oncol 3, 5–8. 19383361 10.1016/j.molonc.2008.11.001 PMC5527865

[mol212452-bib-0004] Celis JE and Gago JM (2014) Shaping science policy in Europe. Mol Oncol 8, 447–457. 24726457 10.1016/j.molonc.2014.03.013 PMC5528628

[mol212452-bib-0005] Celis JE and Pavalski D (2017) A mission‐oriented approach to cancer in Europe: a joint mission/vision 2030. Mol Oncol 11, 1661–1672. 29024497 10.1002/1878-0261.12143 PMC5709667

[mol212452-bib-0006] David P (2007) The Historical Origin of ‘Open Science’ ‐ An Essay on Patronage, Reputation and Common Agency Contracting in the Scientific Revolution. Stanford, CA: Stanford Institute for Economic Policy Research.

[mol212452-bib-0007] De Lorenzo F and Apostolidis K (2019) The European Cancer Patient Coalition and its central role in connecting stakeholders to advance patient‐centric solutions in the mission on cancer. Mol Oncol 13, 653–666. 10.1002/1878-0261.12448 PMC6396363 30657631

[mol212452-bib-0008] Eggermont AM , Apolone G , Baumann M , Caldas C , Celis JE , de Lorenzo F , Ernberg I , Ringborg U , Rowell J , Tabernero J *et al* (2019) Cancer Core Europe: a translational research infrastructure for a European mission on cancer. Mol Oncol 13, 521–527. 10.1002/1878-0261.12447 PMC6396377 30657633

[mol212452-bib-0009] Eggermont AM , Caldas C , Ringborg U , Medema R , Tabernero J and Wiestler O (2014) Cancer Core Europe: a consortium to address the cancer care‐cancer research continuum challenge. Eur J Cancer 50, 2745–2746. 25263570 10.1016/j.ejca.2014.07.025

[mol212452-bib-0010] Ernberg I (2019) Education aimed at increasing international collaboration and decreasing inequalities. Mol Oncol 13, 648–652. 10.1002/1878-0261.12460 PMC6396351 30677237

[mol212452-bib-0011] Ernst RR (2003) The responsibility of scientists, a European view. Angew Chem Int Ed 42, 4434–4439. 10.1002/anie.200330065 14520738

[mol212452-bib-0012] Ernst D and Kim L (2002) Global production networks, knowledge diffusion, and local capability formation. Res Policy 31, 1417–1429.

[mol212452-bib-0013] European Academy of Cancer Sciences (2019) A report from a conference jointly organised by the European Academy of Cancer Sciences and the Pontifical Academy of Sciences, The Vatican, November 16–17, 2018. Mol Oncol 13, 511–516. 10.1002/1878-0261.12436 PMC6396569 30811840

[mol212452-bib-0014] Ferlay J , Colombet M , Soerjomataram I , Dyba T , Randi G , Bettio M , Gavin A , Visser O and Bray F (2018) Cancer incidence and mortality patterns in Europe: estimates for 40 countries and 25 major cancers in 2018. Eur J Cancer 103, 356–387. 30100160 10.1016/j.ejca.2018.07.005

[mol212452-bib-0015] Forman D , Bauld L , Bonanni B , Brenner H , Brown K , Dillner J , Kampman E , Manczuk M , Riboli E , Steindorf K *et al* (2018) Time for a European initiative for research to prevent cancer: a manifesto for Cancer Prevention Europe (CPE). Journal of Cancer Policy 17, 15–23.

[mol212452-bib-0016] Fricker J (2010) The unified approach: meeting the cancer challenges of the future. Mol Oncol 4, 1–8.

[mol212452-bib-0017] Gannon F (2017) The ERC: from before then to anniversary celebrations. EMBO Rep 11, 1873–1874. 10.15252/embr.201745025 PMC5666599 29021205

[mol212452-bib-0018] Heitor M (2015a) How far university global partnerships may facilitate a new era of international affairs and foster political and economic relations? Technol Forecast Soc Chang 95, 276–293.

[mol212452-bib-0019] Heitor M (2015b) Science policy for an increasingly diverging Europe. J Res Policy Eval 2, 16–37.

[mol212452-bib-0020] Heitor MV and Horta H (2014) Democratizing higher education and access to science: the Portuguese reform 2006‐2010. High Educ Policy 27, 239–257.

[mol212452-bib-0021] Heitor M , Horta H and Mendonça J (2015) Developing human capital and research capacity: science policies promoting brain gain. Technol Forecast Soc Chang 82, 6–22.

[mol212452-bib-0022] König T (2017) The European Research Council. Hoboken, NJ: Wiley.

[mol212452-bib-0023] Oberst S (2019) Bridging research and clinical care – the comprehensive cancer centre. Mol Oncol 13, 614–618. 10.1002/1878-0261.12442 PMC6396367 30628155

[mol212452-bib-0024] Ringborg U (2008) The Stockholm declaration. Mol Oncol 2, 10–11. 19383323 10.1016/j.molonc.2008.03.004 PMC5527785

[mol212452-bib-0025] Saghatchian M , Hummel H , Otter R , de Valeriola D , Van Harten W , Paradiso A , Koot B , Ringborg U and Tursz T (2008) Towards quality, comprehensiveness and excellence. The accreditation project of the Organisation of European Cancer Institutes (OECI). Organisation of European Cancer Institutes. Tumori 94, 164–171. 18564602 10.1177/030089160809400206

[mol212452-bib-0026] Saul H (2008) Cancer research in Europe: what next? Eur J Cancer 44, 2087–2091.

[mol212452-bib-0027] Van de Loo JW , Trzaska D , Berkouk K , Vidal M and Akli R (2012) Emphasizing the European Union's Commitment to Cancer Research: a helicopter view of the Seventh Framework Programme for Research and Technological Development. Oncologist 17, 26–32. 23104172 10.1634/theoncologist.2012-0327 PMC3481902

[mol212452-bib-0028] Ziman J (1968) Public Knowledge: The Social Dimension of Science. Cambridge: Cambridge University Press.

[mol212452-bib-0029] Ziman J (1978) Reliable Knowledge: An Exploration of the Grounds for Belief in Science. Cambridge: Cambridge University Press.

[mol212452-bib-0030] Ziman J (2000) Real Science: What it is, and What it Means. Cambridge: Cambridge University Press.

